# Supplementary material for: Polymorphisms in rs2069845 are associated with IL-6 and soluble IL-6 receptor levels during total joint replacement
Source: PLoS One. 2025 May 15;20(5):e0312985. doi: 10.1371/journal.pone.0312985 (PMC12080868; doi:10.1371/journal.pone.0312985)
Supplement: Attachment — Submitted filename: Anderson et al - response to reviewer comments.docx [file pone.0312985.s003.docx]

We thank the reviewers for their helpful feedback. We have organized our responses to each of the comments below and wherever appropriate, give a description of the in text changes that have been made to address their suggestions.

| Reviewer comment/suggest | Author response | Change in text |
| --- | --- | --- |
| The language/grammar/syntax needs to be edited in several areas especially the introduction and some of the methods as it was hard to understand in some areas. | We reorganized the introduction, particularly the section on IL-6/sIL-6r signaling, which was likely too complicated. We have also provided more detailed context on this signaling pathway and its implications in osteolysis in the discussion. | The introduction has been re-written to focus our mechanistic description. Hopefully, this improves the readability. |
| Please italicize the gene throughout the manuscript. |  | When referring to the genes of interest, we’ve updated the text to italicize. |
| What was the biological rationale for the selection of these SNPs? | We focused on SNPs related to IL-6 based on our previously published study that noted a link between early IL-6 levels and surgical outcomes. We therefore searched the literature for studies with IL-6 signaling implications. | We’ve updated the introduction to more specifically outline our SNP selection process (lines 91 to 98). |
| Please insert a paragraph of sample size in the methodology section, please include the study power and a short description of how it was calculated. | This paragraph is present within the methods in a sub-section section titled *A prior power analysis.* | The paragraph has been updated in response to review 2’s question. |
| How much is the MAF? Please include it in the method section | Minor allele frequency  Pulled from the ALFA database using National Library of Medicine bdSNP. | The MAF data was added to the methods section (line no. 154). |
| The Hardy-Weinberg equilibrium should be performed considering the control and subjects population | We ran our allele frequencies through the HW test package in R and found that all met the expectations of the HW principle. | The methods section was updated to note this and the p-value results are now included in table 1. |
| In discussion, please provide focused description on the effects of the SNPs |  | Based on this comment and those of reviewer two, we have performed extensive re-writes of the discussion to include more background description. |
| Please, move the paragraph of ethical approval with the IRB number from the acknowledgment section to the methodology section. | Thank you for this suggestion | This sentence in question has been moved to the methods (line nos. 103-104). |
|  |  |  |
| Line no. 62 “several cytokines …… late-stage biomarkers” authors should mention the other cytokines. | Good suggestion. We’ve added reference to the cytokines that have been studied and have some degree of consistency in reporting significant associations with osteolysis. | Added: “including IL-6, TNF, IL-1β, and IL-8” |
| why rs2069845 has been used by the author (reported for leprosy)? Materials and method: | We wanted to include a SNP that was associated with IL-6-related inflammatory reactions. Our justification stems from our previous longitudinal biomarker study, which found that patients that would eventually develop osteoporosis had a greater IL-6 response following joint replacement. | We have updated the introduction, per reviewer’s 1’s comments, to provide better justification. |
| What sample size was predicted when the authors performed power analysis? Please mention the test details, sample size, and version of the Quanto power calculation tool. | We have updated our power analysis section. This includes more details about the analysis we ran in Quanto. We also initially performed a cruder analysis using a paired comparisons design, which we have also described. | The power analysis section in the methods has been re-written with more detail (lines 108 to 122). |
| Three SNPs are mentioned in the line no. 85 of the introduction, but it has been changed to four SNPs in line no. 103. | Apologies, this was a typo. | This sentence has been updated. |
| Why age and gender-matched control has not been taken. | In this study, our primary hypothesis was related to the change in IL-6 and sIL-6r after total joint replacement surgery. It isn’t clear with this study design, what the control population would be, as patients who did not undergo surgery would not have a response to surgery. | Acknowledged now as a limitation in the final discussion paragraph, starting at line no. 343. |
| Line no. 116 - initials of Lead investigators are not necessary. |  | Removed |
| Rewrite IL6-R (line no. 132) as IL-6r | Thank you for catching this. | Edited. |
| The author should include either an abbreviation table in the beginning or expand the acronyms used in the manuscript wherever it is mentioned for the First time for example, TKR (Line no. 157); and THR (Line no. 157). | Apologies for the oversight. | We’ve updated the methods to define the abbreviations TKR and THR (line nos. 126-127). We’ve also updated the footer to table 1 to define these abbreviations again. |
| Are there SNPs reported from other cytokine genes related to bone resorption after TJR? | There are other cytokine SNPs that have been linked to osteolysis, which is caused by aberrant resorption. | We updated our discussion to include references to some of the papers that have linked cytokine SNPs to bone resorption or orthopedic implant failure. Due to space constraints, we did not summarize the literature related to non-cytokine SNPs or those linked to osteolysis around dental implants but do point the readers to a nice review article on this topic (lines: 323 to 342). |
| What are the other biomarkers/proteins that influence IL6/IL6 receptor binding? | Is the reviewer asking about gp130? While gp130 is a critical IL-6 signaling mediator, it is a transmembrane protein and therefore not a good biomarker.  While it does not directly influence the binding of IL6 to sIL6r or gp130, RANKL is a critical signaling involved in IL-6 induced osteoclastogenesis. | We’ve added information about the role of RANKL in IL-6 signaling and osteolysis and included reference to the link between SNPs in gp130 and inflammatory diseases (lines: 309 to 322). |
| Besides implant material, what other possible factors that cause intrinsic sensitivity of patients also need to be mentioned? | Is the reviewer referring to the statement in paragraph 2 of the discussion? We have updated the discussion to include reference to some analyses that have attempted to summarize possible factors contributing to variability in the response to implants. | Some of these factors include sex, activity, and comorbid conditions, which have been described and are now summarized in the discussion (lines 323 to 328). |
| Legends for figures 1 and 2 should have the p-value. | The legends for each figure show all p-values for the two-way ANOVA results (seen in panels c and f in each figure). For the timepoint comparisons, we are not always able to perform one-way ANOVAs due to the low sample size for some of the rarer alleles. We do show paired differences between two groups. | Previously we only indicated when the p-value was less than 0.05. We have updated the figures now to show the specific p-values. |
| Provide footnote for tables 2,3, 4, and 5 |  | Footnotes have been added to the tables to provide information about the abbreviations used (Table 1). There are no additional tables within the manuscript draft. |
| Authors need to give details of statistical tests in Tables for clarity | The only table is a demographic data (Table 1). We have updated based on reviewer 1’s comments to reference HWE data now. | Table 1 now includes reference to the HWE test. |
| Limitations of the study should be incorporated after the discussion. | The last paragraph of the discussion includes reference to what we consider limitations. | See lines 343 to 363. |
| The authors need to improve the resolution of all the figures | The figures are created in Prism and exported at the required 600 dpi. | While updating the figures to include the new p-value descriptions, we re-exported to ensure that they were at the required 300 dpi resolution. |
| Discussion and conclusion require a systematic writing |  | Based on the reviewer comments, we have updated the discussion to include several new topics and citations. Hopefully this editing has helped clarify. |
