# Supplementary material for: Polymorphisms in rs2069845 are associated with IL-6 and soluble IL-6 receptor levels during total joint replacement
Source: PLoS One. 2025 May 15;20(5):e0312985. doi: 10.1371/journal.pone.0312985 (PMC12080868; doi:10.1371/journal.pone.0312985)
Supplement: Attachment — Submitted filename: Second revision reviewer comments.docx [file pone.0312985.s004.docx]

| Reviewer 1 | | |
| --- | --- | --- |
| Thanks alot for your efforts, in doing all required modifications All comments have been addressed, and the manuscript is valid or publication in the current form | Thank you for your review. | No changes. |
|  |  |  |
| Reviewer 2 | | |
| In line 153, there is a typo that needs to be corrected. Please change "bdSNP" to "dbSNP.". | Thank you. | Edited as requested |
| The minor allele frequency in line 154 should be rechecked using the 1000 Genomes database or dbSNP for the European population rather than the global population, followed by SNP analysis. For instance, the ref. allele “C” of rs4537545 has a frequency of 0.592611 in the European population (rs4537545 RefSNP Report - dbSNP - NCBI), whereas in figure-3, very few individuals are homozygous CC, with low mean pre-operative IL-6 level. Therefore, normally, SNP-based studies or analyses should not be done with a low sample size. | Per the reviewer’s suggestion, we have updated the stated MAF to pull from the 1000 Genomics database. The methods now refer to the global and European MAFs from 1000 genomics.  We acknowledge the reviewer’s point about the low sample size and have referred to this as a limitation. Our IRB limited our ability to continue recruitment after reaching our necessary sample size. | Updated the reported MAF values in the methods – lines 178-183. |
| What tests are used for Hardy-Weinberg equilibrium analysis of SNPs in R-Studio? Please mention the test name in the methodology section. | We used the likelihood-ratio test in R-studio (HW.lrtest). We also ran the exact test (HW.exacttest) and the results were very similar. | Updated the methods (lines 181-182) and the table legend in table 1. |
| The SNPs discussed in the article are located within introns (rs4537545 RefSNP Report - dbSNP - NCBI ; rs2069845 RefSNP Report - dbSNP - NCBI) and missense variant (rs2228145 RefSNP Report - dbSNP - NCBI), therefore, to strengthen these findings, the authors should conduct in-silico analyses to demonstrate how these SNPs regulate IL6 levels. Authors can utilize various online tools available on the UCSC browser for this purpose. This approach would also differentiate their manuscript from previous studies focusing on these variants (e.g., reference 25). | We appreciate the suggestion as this was not something that we considered. We investigated several in silico tools to determine what would work best. Many of the UCSC tools, including PolyPhen-2 and SIFT didn’t appear to work to work for two of the SNPs, which were intronic. Therefore, we opted to use the gnomAD browser tool, which is better able to accommodate the intron mutations. We now provide the in silico predictive values from the CADD and phyloP databases in table 1. | The results are updated and described in the methods: line 184-193. |
